# Side effects of topical atropine 0.05% compared to 0.01% for myopia control in German school children: a pilot study

**DOI:** 10.1007/s10792-021-01755-8

**Published:** 2021-02-25

**Authors:** Lutz Joachimsen, Navid Farassat, Tim Bleul, Daniel Böhringer, Wolf A. Lagrèze, Michael Reich

**Affiliations:** Eye Center, Medical Center - University of Freiburg, Faculty of Medicine, University of Freiburg, Killianstrasse 5, 79106 Freiburg im Breisgau, Germany

**Keywords:** Myopia, Children, Low-dose atropine, Side effects, Europe, Caucasian

## Abstract

**Purpose:**

Based on findings of the Asian low-concentration atropine for myopia progression study, a concentration of 0.05% has been proposed as a good compromise between safety and efficacy for myopia control. However, no data on side effects have been published so far in Caucasian children receiving this dose.

**Methods:**

Prior to commencement of bilateral atropine treatment with 0.05% atropine, 19 myopic children aged 5 to 15 years were treated in only one eye at bedtime leaving the other eye as a control. Pupil size, accommodation amplitude and near visual acuity were measured at 10:00 a.m. the next day and compared to the untreated contralateral control eye. The results were then compared to a cohort of 18 children whose treatment with 0.01% atropine commenced in a similar fashion.

**Results:**

Twelve children (63%) reported visual impairment or reading difficulties. Anisocoria was 2.9 ± 1.1 mm. In comparison, 0.01% atropine led to a significantly less anisocoria of 0.8 ± 0.7 mm (*p* < 0.0001). Accommodation was decreased by − 4.2 ± 3.8 D in 0.05% atropine treated eyes, whereas 0.01% atropine induced hypoaccommodation of − 0.05 ± 2.5 D (*p* < 0.01). Near visual acuity was not significantly reduced in eyes treated with 0.05% atropine compared to 0.01% atropine (*p* = 0.26).

**Conclusion:**

Compared to 0.01%, our data indicate stronger more relevant side effects of 0.05% topical atropine in young Caucasian children with progressive myopia as recently reported in Asian children, potentially compromising acceptance and compliance.

## Introduction

Myopia is the most common ocular anomaly manifesting during the first two decades of life. It now affects about 1.95 billion individuals worldwide with 0.28 billion suffering high myopia [1]. Prevalence of more than 80% has been repeatedly reported from East Asian countries [2]. Population-based studies from Europe indicate a current rate of 47% in young adults with rising incidences over recent generations [3]. This development is likely to result from changing environmental factors [4], mainly increased near-work and less outdoor time leading to a lack of environmental light exposure which is considered necessary to prevent myopia via a dopamine-mediated mechanism [5].

Myopia does not only impose high costs on health systems and societies [6]; it is also a risk factor for secondary degenerative eye diseases such as glaucoma, retinal detachment or macular degeneration [7], potentially leading to irreversible sight impairment, especially in high myopia [8]. Myopia usually commences during primary school and progresses until a mean age of 16, but rarely beyond an age of 25 years [9]. It is desirable to influence its progression from the very beginning, i.e., during primary school, when progression is fastest [10].

Apart from sufficient outdoor time [11] and optical aids correcting for peripheral retinal defocus [12], topical atropine is the mainstay of myopia control [13, 14]. While this is known for more than a century [14], it was until the pivotal ATOM-2 (atropine for the treatment of myopia) RCT from Singapore that has sparked a tremendous worldwide interest in low-dose atropine (LDA) therapy [15]. In the meanwhile, it became a standard treatment in many countries favoring a concentration of 0.01%. Subsequently, the LAMP study (low-concentration atropine for myopia progression) from Hong Kong addressed safety and efficacy in more subtle concentration increments of 0.01%, 0.025% and 0.05% as compared to placebo. 0.05% was found to be most effective with a sufficient safety profile regarding side effects such as mydriasis and hypoaccommodation leading to light sensitivity and reading difficulties [16].

Before adapting the suggested treatment regime of the LAMP study to non-Asian populations, more information is needed on its safety profile and side effects. While 0.01% has become a widely accepted use and is recommended in national guidelines in Europe, some parents ask for 0.05% instead, based on the finding of the LAMP study. We therefore documented side effects in a small group of 20 German children whose LDA therapy was begun with 0.05% atropine.

## Methods

### Study design and population

This is a cross-sectional, single-center, observational case series conducted between September 2019 and March 2020 on children with confirmed myopia greater than − 1.0 D. The study was approved by the institutional Ethics Committee of the University of Freiburg (Institutional Review Board Approval University of Freiburg #287/16) and adhered to the tenets of the Declaration of Helsinki. Informed consent for further use of collected data was obtained by the parents at the first presentation.

### Inclusion and exclusion criteria

Inclusion criteria were an age of 5 to 17 years and an annual myopic progression of greater than 0.5 D. Exclusion criteria were non-Caucasian origin, syndromic progressive myopia, anisocoria over 0.5 mm or any known eye disease as well as any previous treatments for myopia control.

### Medication

Unpreserved 0.05% atropine eyedrops in single dose units were supplied by a pharmacy (Berg-Apotheke Tecklenburg, Germany). Atropine concentrations were reconfirmed by the pharmacy of our institution using a validated liquid chromatography method (ReproSil-Pur Basic column C18, Dr. Maisch, Ammerbuch-Entringen, Germany). The hydrolysis products tropic acid and tropine were detected by mass spectrometry (Bruker QTOF, Karlsruhe, Germany). For quantification, an external calibration with 0.05% atropine sulfate solution was used (regression coefficient 0.999, precision < 2%). To prove the durability of this preparation, the eyedrops from the same charge were stored at room temperature for 9 months showing a slight increase in pH from 4.4 to 4.5 and a decrease in atropine sulfate concentration from 100 to 94%.

### Data collection

Before treatment, non-cycloplegic automated refraction (RM-8900, Topcon, Tokyo, Japan) followed by subjective refraction was performed for future analysis of progression. Myopia progression during the last year and iris color (light or dark) were documented. The children and their parents were instructed to apply one eyedrop once before bedtime to just one eye leaving the other eye as an intraindividual control.

The next morning at 10:00 a.m., as a representative time during the school morning, pupil size, accommodation amplitude and near visual acuity in both eyes, the treated and the non-treated one, were measured. The pupil size was measured in photopic conditions with the cross-lines of the eyepiece of a manual Goldmann perimeter (Haag-Streit, Bern, Switzerland) illuminated with 10 cd/m^2^. The accommodation near point was determined by dynamic retinoscopy averaging three measurements: The patients were asked to read optotypes, while the retinoscope (Heine Beta 200, Gilching, Germany) was continuously approximated. The changeover from the fundus red flickering reflex to a “with movement” was defined as the near point of accommodation. Near vision was tested with Landolt optotypes (C test for near vision, Oculus, Wetzlar, Germany) and best-corrected distance refraction in 30 cm. For the statistical evaluation, the decimal visual acuity was converted to logMAR. Eyes were checked for topical side effects via slit lamp examination. A self-designed questionnaire was used to ask for symptoms and side effects of atropine use. The children and their parents were asked whether they had noticed any changes after the eyedrops. Specifically, they were asked about “visual impairment,” “reading difficulties,” “burning sensation,” “diplopia,” “light sensitivity” or “glare.” Answers were classified as “no problem” or “problem mentioned on demand or described by the patient herself/himself.” Commencement of bilateral treatment was scheduled for the day after.

### Side effects of 0.05% compared to 0.01% atropine

To compare side effects of 0.05% with 0.01% atropine, we used data from children, previously examined in our institution in a similar fashion at the beginning of a therapy with 0.01% atropine [17]. The secondary arm of the previous study [17] addressed the issue of side effects and included 20 patients who started 0.01% atropine in one eye only the evening before and presented the next day for an ophthalmic examination. The extent of anisocoria, change of accommodation amplitude and near visual acuity measured at 10 a.m. were obtained in 18 of these 20 children. Data of these 18 children were used as the comparison group for the present study.

### Statistical methods

Statistical analysis was performed using SPSS V20.0 and GraphPad Prism 6 (GraphPad Software, Inc., La Jolla, CA, USA). A probability (*p*) value of < 0.05 was considered statistically significant. For descriptive data analysis, the mean and standard deviation (SD) were calculated. Box–whisker plots (Tukey) were performed. To compare between two groups in a nonparametric way, the Mann–Whitney U test was used. To compare the effect of 0.05% atropine on anisocoria, change of accommodation amplitude and near visual acuity to the results of our previous published data of a cohort treated with 0.01% atropine [17], a univariate linear model (ANOVA) was performed adjusting for age, sex and iris color.

## Results

### Patients’ characteristics

After excluding one child from the data analysis due to late arrival at the follow-up examination, data from nineteen children (male/female 12/7) were included for analysis. Detailed information about age, sex, iris color, refraction and visual acuity of the study cohort compared to the cohort of our previous published data of children treated with 0.01% atropine [17] is illustrated in Table 1.

**Table 1:** Demographics of cohort treated with 0.01% atropine previously published by Joachimsen et al. [17] compared to new cohort treated with 0.05% atropine before treatment. Mann–Whitney U test: *p = 0.034, **p = 0.036

		0.01% atropine	0.05% atropine
Number of children		18	19
Age (years)		9.6 ± 2.0*	10.8 ± 2.1*
Sex (male/female)		8/10	12/7
Iris color (light/dark)		9/9	9/10
Refraction (D)			
	*Treated eye*	− 3.04 ± 1.54**	− 4.61 ± 2.10**
	*Untreated eye*	− 3.08 ± 2.68	− 4.34 ± 2.01
Visual acuity (logMAR)			
	*Treated eye*	− 0.04 ± 0.09	− 0.06 ± 0.12
	*Untreated eye*	− 0.04 ± 0.10	− 0.03 ± 0.12

### Effect of 0.05% atropine on pupil size, accommodation amplitude and near visual acuity compared to the untreated fellow eye

**Fig. 1:**
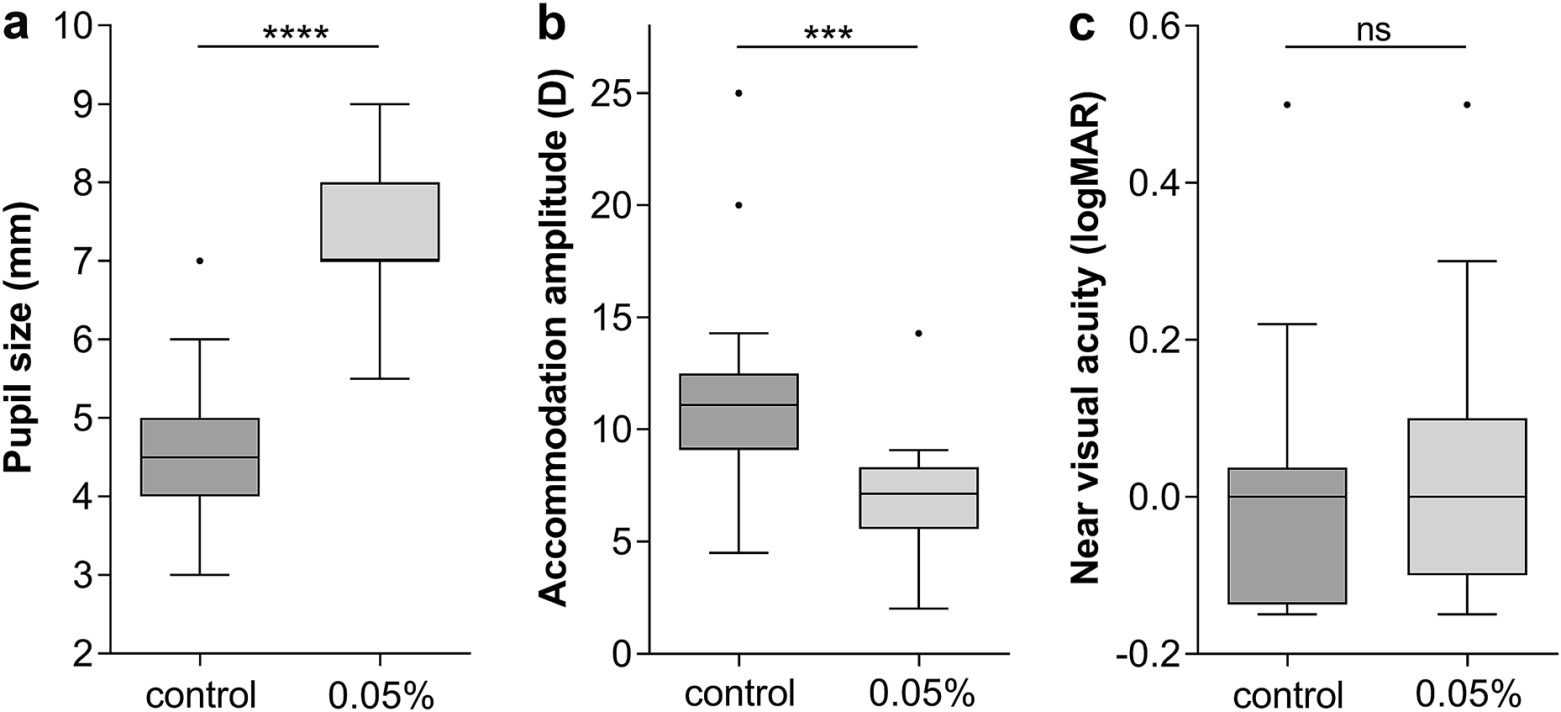
Box–whisker plots (Tukey) showing differences between untreated eyes (control) and treated eyes (0.05%). **a** Pupil size, **b** accommodation amplitude and **c** near visual acuity measured at 10:00 a.m. after unilateral application of 0.05% atropine at the previous evening. For comparison, Mann–Whitney U test was performed, ****p < 0.0001, ***p < 0.001, ns *p* = 0.24

Pupil sizes and accommodation amplitudes of eyes treated with 0.05% topical atropine and untreated eyes were significantly different (pupil size: 7.4 ± 0.9 mm compared to 4.6 ± 1.0 mm, *p* < 0.0001, Fig. 1a; accommodation amplitude: 7.0 ± 2.6 D compared to 11.2 ± 4.9 D, *p* < 0.001, Fig. 1b). However, near vision was almost unaffected in the treated compared to the untreated eyes (0.04 ± 0.16 logMAR compared to − 0.01 ± 0.16 logMAR, *p* = 0.24, Fig. 1c). Anisocoria, as well as difference of accommodation amplitude, respectively, near vision, between treated and untreated eye was not influenced by iris color (all *p* > 0.54). No topical side effects such as conjunctival redness or other changes were observed. Twelve children (63%, light/dark iris color 3/9) complained of visual impairment or reading difficulties, and seven (37%, light/dark iris color 6/1) had no symptoms related to the therapy. None of the children reported eye burning, diplopia, light sensitivity or glare.

### Effect of 0.05% atropine compared to 0.01% atropine on pupil size, accommodation amplitude and near visual acuity

**Fig. 2:**
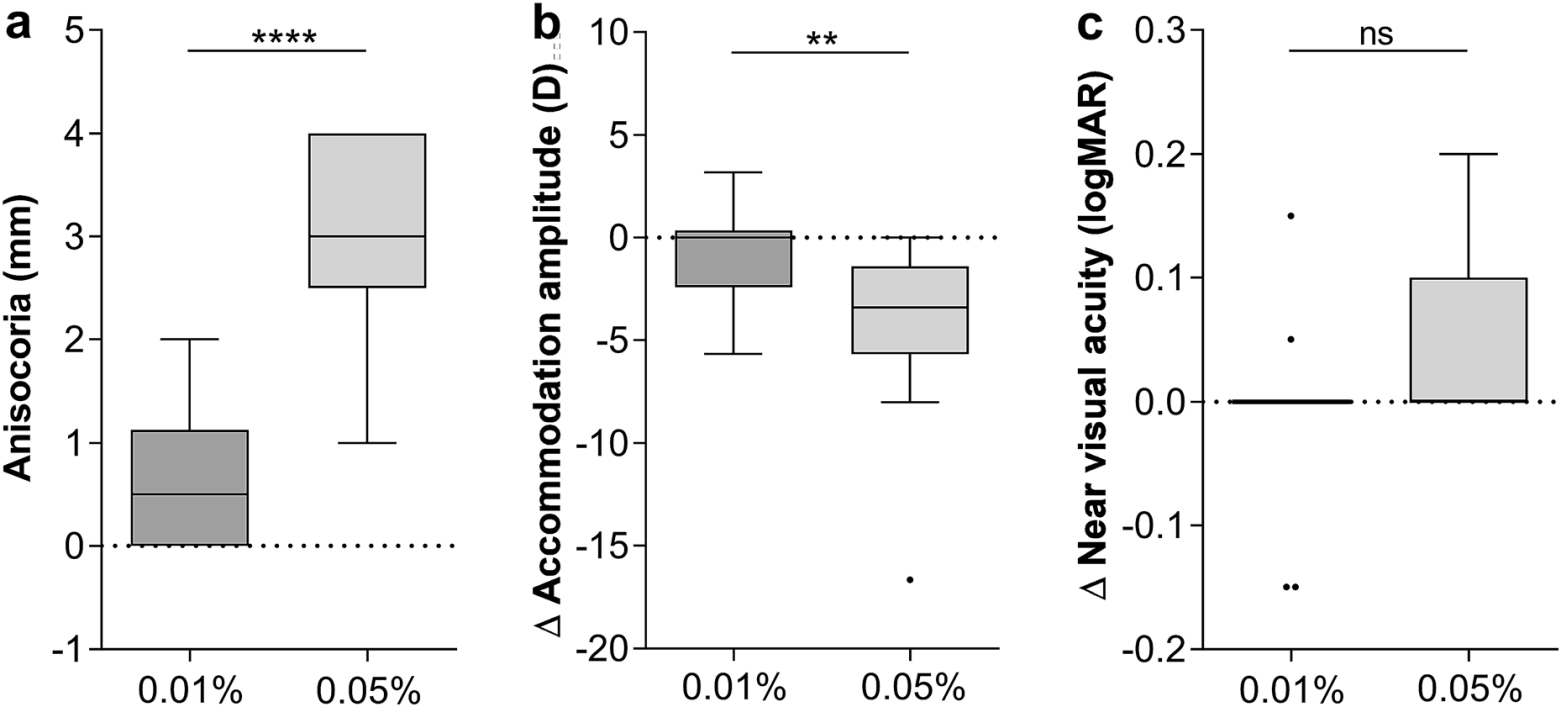
Box–whisker plots (Tukey) comparing side effects of 0.01% and 0.05% topical atropine as interocular difference (Δ) between treated and untreated eye in **a** pupil size, **b** accommodation amplitude and **c** near visual acuity, measured at 10:00 a.m. after unilateral application of 0.01% (*n* = 18, data previously published by Joachimsen et al. [17]) and 0.05% atropine (*n* = 19) on the previous evening. Age-, gender- and iris color-adjusted univariate linear model (ANOVA) was used for analysis. In all three analyses, the three covariates age, gender and iris color showed a p value of > 0.30. ****p < 0.0001, **p < 0.01, ns *p* = 0.26

Compared to our previously published data of a cohort treated with 0.01% atropine [17] and adjusting for age, iris color and sex, children treated with 0.05% atropine showed significant differences in anisocoria (2.9 ± 1.1 mm compared to 0.8 ± 0.7 mm, *p* < 0.0001, Fig. 2a) and loss of accommodation amplitude (− 4.2 ± 3.8 D compared to − 0.05 ± 2.5 D, *p* < 0.01, Fig. 2b). Regarding loss of near vision, no difference between both cohorts could be detected (0.05 ± 0.06 logMAR compared to − 0.01 ± 0.06 logMAR, *p* = 0.26, Fig. 2c).

## Discussion

In this study, we report short-term ocular side effects and adverse events of topical 0.05% atropine prescribed for myopia control in 19 Caucasian children. They were more pronounced as described in the LAMP study with 438 children from Hong Kong [16, 18]. While in the LAMP study [16] photopic pupil size was increased by 1.1 mm after 4 months of therapy with 0.05% atropine, an anisocoria of 2.9 mm could be detected in our group. Accommodation amplitude decreased by − 4.2 D, compared to − 2.4 D being described in the LAMP study after 4 months of therapy. Even though these side effects are not reflected in reduction in near visual acuity in both the LAMP and our much smaller cohort, 63% of our children described visual impairment or reading difficulties. Therefore, our observations suggest more pronounced side effects of a topical therapy with 0.05% atropine in Caucasian children than observed in an Asian population. This is in line with the results of Cooper et al. who compared three different doses of topical atropine (0.012%, 0.025% and 0.05%) in US-American children with brown irises. They defined 0.02% as a threshold dose for relevant side effects, like blurred vision, fatigue, diplopia, difficulty in concentrating, sunlight sensitivity or glare [19].

Safety-related data on LDA are still rare in Caucasian children. Joachimsen et al. observed a pupil dilation of 1 mm in children with 0.01% atropine therapy and negligible hypoaccomodation without an effect on near vision [17]. Sacchi et al. reported photophobia in 9.6% as only detectable adverse event with 0.01% atropine, and data on pupil size were not presented [20]. Diaz-Llopis and Pinazo-Durán reported that in a 5-year observation period 2% of children treated with 0.01% atropine discontinued therapy due to photophobia, difficulties in reading, mydriasis or headache [21]. Comparing the side effects in our 19 German school children being treated with 0.05% atropine to our previously published data of side effects in 18 children after the use of 0.01% atropine [17], we found that 0.05% atropine induced significantly more anisocoria (2.9 mm compared to 0.8 mm) and loss of accommodation amplitude (loss of 4.2 D compared to 0.05 D, Fig. 2). Hence, the discrepancy in anisocoria and hypoaccommodation was more pronounced in our population than in the LAMP trial showing a difference between both concentrations in pupil size of 0.8 mm and accommodation 1.9 D. Near visual acuity showed no clear differences among both concentrations in both cohorts. Since mydriasis is expected to lead not only to glare sensation but also to higher optical aberration errors especially in a pupil diameters > 3 mm [22], it did not reach a threshold leading to reduction in near visual acuity.

It remains yet unknown as to why the side effects were more pronounced in our non-Asian cohort with high variation in iris color. Up to now, one can only speculate on the role of ocular melanin and the affinity of atropine for melanin [23]. This is in line with data from Nishiyama et al., who found no side effects 2 weeks after 0.01% atropine in Japanese children aged between 6 and 12 years [24]. Probably due to our small sample size, an influence of iris color on the probability of side effects could not be detected. Further studies with larger non-Asian or mixed cohorts would be desirable to further investigate this issue.

Beside the small sample size, a major limitation of this report, its retrospective nature is another limitation, although the data were collected consecutively. Hence, we could not ideally match the cohort treated with 0.05% atropine to the previously published cohort treated with 0.01% atropine in terms of age and refraction. Nevertheless, a univariate linear model (ANOVA) adjusting for age, sex and iris color excluded the statistical influences of these parameters when comparing both cohorts. Another relevant limitation is the short follow-up period of just one day, because parents were referred for bilateral treatment, which we did not want to defer for ethical reasons for a longer period of time. Therefore, we cannot draw any conclusion based on an intraindividual comparison on more long-term side effects of 0.05% atropine. Any further study would fall under the auspices of the German Medicines Law requiring extensive funding and approval measures. Finally, it shall be mentioned that masking of the treated eye was not possible due to the obvious anisocoria, and bias during the examination by both the examiner and the patient cannot be ruled out.

In summary, our data indicate stronger side effects of 0.05% topical atropine preventing progressive myopia in European Caucasian school children as recently reported in Asian children. From our data, it can be deduced that an individual estimation of the benefits and side effects of different dosages of topical atropine must be carefully evaluated in each individual case. However, in clinical practice of pharmacologic myopia control several questions remain open, e.g., the minimally effective concentration, the yet not completed understood mode of action, the duration of therapy and the selection of patients which profit most. Since efficacy data were collected only in Asian populations, other randomized clinical trials in a Caucasian population are necessary and are currently either in preparation or ongoing.

## Data Availability

More data if necessary are available from the corresponding author on reasonable request.
